# Effects of Jasmonic Acid on Stress Response and Quality Formation in Vegetable Crops and Their Underlying Molecular Mechanisms

**DOI:** 10.3390/plants13111557

**Published:** 2024-06-04

**Authors:** Jiaqi Wu, Yangyang Chen, Yujie Xu, Yahong An, Zhenzhu Hu, Aisheng Xiong, Guanglong Wang

**Affiliations:** 1 School of Life Science and Food Engineering, Huaiyin Institute of Technology, Huaian 223003, China; wujiaqi011015@163.com (J.W.); 17834830201@163.com (Y.C.); 13236206281@163.com (Y.X.); yhan@hyit.edu.cn (Y.A.); huzhenzhu1001@hyit.edu.cn (Z.H.); 2 Jiangsu Provincial Agricultural Green and Low Carbon Production Technology Engineering Research Center, Huaian 223003, China; 3 State Key Laboratory of Crop Genetics & Germplasm Enhancement and Utilization, College of Horticulture, Nanjing Agricultural University, Nanjing 210095, China

**Keywords:** JA, vegetable, quality, JA accumulation, stress response, molecular mechanisms

## Abstract

The plant hormone jasmonic acid plays an important role in plant growth and development, participating in many physiological processes, such as plant disease resistance, stress resistance, organ development, root growth, and flowering. With the improvement in living standards, people have higher requirements regarding the quality of vegetables. However, during the growth process of vegetables, they are often attacked by pests and diseases and undergo abiotic stresses, resulting in their growth restriction and decreases in their yield and quality. Therefore, people have found many ways to regulate the growth and quality of vegetable crops. In recent years, in addition to the role that JA plays in stress response and resistance, it has been found to have a regulatory effect on crop quality. Therefore, this study aims to review the jasmonic acid accumulation patterns during various physiological processes and its potential role in vegetable development and quality formation, as well as the underlying molecular mechanisms. The information provided in this manuscript sheds new light on the improvements in vegetable yield and quality.

## 1. Introduction

With the improvement in living standards, people have higher requirements regarding the quality of vegetables. As an important component of the daily diet, vegetables are rich in nutrients such as sugars, pigments, vitamins, minerals, and dietary fiber, which are crucial for human health [1]. Vegetables with comprehensive and balanced nutrition to meet the various functional needs of the body and maintain normal life activities are more popular. During the growth process of vegetables, they are often invaded by pests and diseases and subjected to abiotic stresses, inhibiting their growth and leading to decreases in their yields and a decline in their quality. Therefore, it is necessary to maintain, regulate, or enhance the original taste and flavor of vegetables during cultivation, storage, and other processes, allowing for a pleasant dining experience. At the same time, people hope that the vegetable market can provide a rich and diverse variety of choices to meet their different nutritional, taste, and dietary needs. By now, many methods have been proposed to regulate, protect, and improve the growth and quality of vegetable crops, including cultivation measures, the application of exogenous plant growth regulators, genetic engineering, and so on [2,3].

Research shows that the plant hormone jasmonic acid (JA) plays an important role in plant growth and development, and it participates in many physiological processes in plants, such as disease resistance, stress resistance, organ development, root growth, and flowering [4,5,6]. In addition to JA’s roles in stress response and resistance, it has also been found to regulate crop quality in recent years. Much progress has been achieved regarding the regulation of JA accumulation and its effects on vegetable growth and quality over the last decades. Therefore, this paper aims to review the jasmonic acid accumulation patterns during various physiological processes and the potential roles in vegetable development and quality formation, as well as the underlying molecular mechanisms. The information provided in this manuscript sheds new light on the improvements in vegetable yield and quality.

## 2. Jasmonic Acid (JA)

Plant hormones play an important role in physiological reactions, such as plant growth and development. Common plant hormones include ethylene (ET), gibberellins (GAs), abscisic acid (ABA), auxin (IAA), jasmonates (JAs), and cytokinin [7].

Jasmonic acid (JA) belongs to a class of fatty acid derivatives containing the basic structure of cyclopentanone. JA is the general name for free jasmonic acid and one of its derivatives, the active precursor substance 12-oxy-phytodienoic acid (OPDA). In addition, its derivatives mainly include jasmonoyl-l-isoleucine (JA-Ile) and methyl jasmonate (MeJA). MeJA was first discovered in 1962 and was extracted from Royal Jasmine by Demole, but it was not until 1980 that Ueda et al. began to notice that this compound has certain growth activity. Jasmonic acid (JA) is an important lipid hormone in plants that is widely found in nature and affects seed germination, root growth, leaf aging, the flowering time, anthocyanin accumulation, trichome development, stamen production, and plant defense responses to biotic and abiotic stresses [8,9,10]. JA in plants is produced by some young and mature tissues and regulates the response of plants to some external environmental stresses (drought, high and low temperatures, and ozone and ultraviolet radiation).

Studies have shown that MeJA can promote terpene biosynthesis in plants and is involved in the stomatal closure of cucumbers [11]. Furthermore, relevant studies have shown that MeJA can regulate the respiratory metabolism, carotenoid synthesis, ethylene synthesis, phenylpropane metabolism, and anthocyanin metabolism of fruit and vegetable products. In addition, it can promote the biosynthesis of plant terpenoids [12]. JA and MeJA have been reported to modulate the accumulation of many secondary metabolites, such as anthocyanins, nicotine, terpenoid indole alkaloids, glucosinolates, and artemisinin [13]. At the same time, many physiological studies have shown that JA has a positive effect on improving plant toxicity caused by heavy metals or trace elements [14,15]. In addition, the active form of JA, JA-Ile, is synthesized in the cytoplasm, and JA-Ile and JA are involved in a variety of physiological activities in plants. After JA-Ile is activated, the resilience of the plant is improved via the control and/or modulation of particular JA-dependent responses [16].

This has led to considerable interest in JA applications for vegetable crops. JA has been applied as a stress hormone to improve plant tolerance to abiotic stress and resistance to necrotrophic pathogens, as well as to control some physiological processes, such as root growth and plant aging [17,18,19].

## 3. JA Biosynthesis and Signaling

### 3.1. JA Biosynthesis

**Figure 1:**
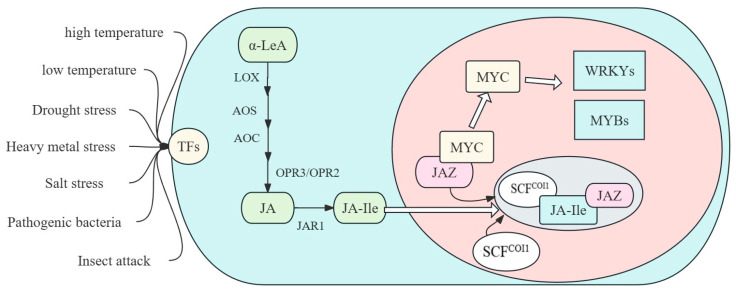
Biosynthetic pathway and signal transduction of JA.

The biosynthesis of JA has been extensively studied in recent decades, especially in Arabidopsis, and at least two pathways have been identified: the α-linolenic acid (18:3) initial octadecane pathway and the hexadecane (16:3) initial hexadecane pathway. The synthetic pathway of JA is carried out in the chloroplasts, peroxisomes, and cytoplasm of plant cells. The essence of its synthesis is a series of enzymatic reactions on the substrate of linolenic acid released by the cell membrane. The synthesis process begins with the entry of linolenic acid into chloroplasts (Figure 1).

First of all, α-LeA produced on the chloroplast membrane is oxidized by lipoxygenase (LOXs) to form the hydroperoxides 9-HPOT and 13-HPOT. Depending on the site of oxygenation, there are two types of LOX: 9-LOX and 13-LOX, which enzymatically form 9-HPOT and 13-HPOT, but only 13-LOX can participate in JA synthesis through the Vick and Zimmermann pathways. Then, 12-oxo-phytodienoic acid (OPDA) is formed under the action of allene-oxidizing synthase and allene-oxidizing cyclase. Then, OPDA is transported via the COMATOSE1 (CTS1) transporter to peroxisome, where it is reduced by OPDA reductase (OPR), ligated to Coenzyme A, and undergoes β-oxidation three times to form JA. Then, JA is transported from the peroxisome to the cytoplasm, where it is catalyzed by jasmonate amino acid synthetase to form biologically active JA-Ile.

JA-Ile can also be catalyzed by jasmonate-isoleucine-12-hydroxylase to produce the less active 12-hydroxy-jasmonate-isoleucine (12-OH-JA-Ile) [20,21]. Other JA metabolic pathways include the hydroxylation of 12-OH-JA or 12-OH-JA-Ile; the sulfonation of 12-OH-JA; and the carboxylation and glucosylation of 12-OH-JA-Ile [22]. In addition, the JA biosynthetic pathway is regulated by substrate availability, positive feedback loops, and tissue specificity, and is involved in Ca^2+^ signal transduction and the regulation of the MAPK cascade [22,23].

### 3.2. JA Signaling

The JA signaling pathway is one of the main mechanisms of plant resistance to various biological and abiotic stresses. In recent years, JA signal transduction has been widely studied and considerable progress has been made. In plants, COI1 (Coronatine insensitive 1) proteins act as receptor proteins for JA signaling and are able to bind to other components to form the SCF-COI1 complex, while causing the degradation of target proteins via ubiquitination [21,24]. The JAZ protein family plays a negative regulatory role in the JA signaling pathway [25]. In the resting state, the JA structural domain in JAZ proteins can interact with MYC2 and inhibit its transcriptional activity [26]. MYC2, a bHLH TF, plays an important role in JA signaling regulation [27,28]. JA signal transduction has the type and specificity of transcription factors (TFs) controlled by repressors such as JAZ and JAV1/JAM. JA-Ile is the most active JA compound and is involved in the regulation of various plant development processes, such as root growth, trichoid formation, and seed germination [22]. Initially, COI1 was thought to be a receptor for JA signal transduction in plant cells, until the discovery of the JAZ protein changed our understanding of the JA signal transduction process [9].

The signal transduction process is mainly divided into three parts: signal generation, signal transduction, and downstream gene expression. Under normal conditions, the level of JA hormone synthesized in plants is relatively low, and the JAZ protein binds to transcription factors such as MYC2 and induces a series of transcriptional suppressors, which work together to inhibit the expressions of JA early-response genes. When plants are subjected to biological or abiotic stress, their JAs or JA-Ile contents increase, which promotes the interaction between the JAZ repressor protein and SCF^COI1^. Meanwhile, F-box protein COI1, as a receptor for JA signal transduction, binds to AtCUL1, AtRbx1, and ASK1/ASK2 to form the SCF-COI1 complex (SCF^COI1^). This interaction causes JAZs to be ubiquitinated as a target protein and degraded through the 26S proteasome pathway, releasing corresponding transcription factors that bind to DNA in the gene cis-acting elements, thereby activating the expressions of jasmonic acid early-response genes [29].

Shan and his colleagues characterized the steps of JA-Ile-induced anthocyanin biosynthesis and the TFs involved in regulation, revealing the specificity among JA-Ile-dependent processes [30]. In 2004, Solano’s lab identified MYC2 as a TF for JA-Ile-induced gene expression [31]. In contrast to the positive regulation of JAZ in plant growth and development, MYC TFs (MYC2, MYC3, and MYC4) negatively regulate gene expression in the cell cycle, thereby inhibiting plant growth. MYC2, as a general switch for the JA signaling pathway, is the best-known TF [32].

## 4. JA Regulation

### 4.1. Growth Accumulation

JA and MeJA have always been considered important signal molecules used to regulate plant resistance gene expression in response to various external biological stresses, and they are effective plant developmental regulators and have certain effects on plant growth and development, tissue maturation and senescence, flowering and flower development, fruit storage, and other processes. Moreover, JA also affects root growth, including primary root inhibition, root regeneration, the promotion of the lateral root structure, and reduction in adventitious root formation [33]. Moreover, JA treatment can reduce plant root elongation by reducing the numbers and sizes of cells [6]. Studies have shown that the physiological response mechanism triggered via JA signaling is mainly carried out through the activation of the antioxidant system, the accumulation of physiological amino acids and soluble sugars, and reductions in stomatal opening and closing activities [34]. Plants have low JA contents, and the changes in the JA contents are different in different plants during their development. In the five developmental stages of carrots, the MeJA contents in the root and leaf were higher in the early stage and lower in the later stage. The JA accumulation trend was the opposite of the growth trend of the carrot root and leaf [35]. The results showed that JA accumulation had an effect on plant growth and development to some extent.

Recent studies have shown that JA is an important flowering-time suppressor. JA regulates flower stamen development under normal conditions and affects root growth and trichoid formation under stress [36]. In addition, JA delays the flowering of other plant species. The JA signaling pathway is involved in the regulation of the flowering times in plants in a concentration-dependent manner. Low JA concentrations promote flower induction, while high JA concentrations inhibit it [37]. In wheat mutant mvp-1 with a deletion-induced nonflowering phenotype compared to the wild type, MeJA accumulation was found in winter wheat during the cold response, and this accumulation declines rapidly when the plant is not adapted to flower-induced growth conditions [38]. This suggests that MeJA may play a role in flower development. In addition, MeJA was applied to spring wheat with insensitivity to vernalization, and the treatment delayed its flowering, which once again proved the role of MeJA in regulating vernalization and flowering time. Similarly, during seed development, CoMYC2, a key transcription factor in the JA signal transduction pathway, can directly interact with three hub genes related to the seed size: *CoCDKB2-3*, *CoCYCB2-3*, and *CoXTH9* [39].

Previous studies have shown that JA is involved in the aging process of plant leaves, and that the JA concentration in aging leaves is much higher than that in young leaves [40,41]. During the whole leaf-aging process, the *LOX1* (lipoxygenase 1), *LOX3*, and *LOX4* expression levels significantly increased, the *AOS* expression level slightly increased, and the *AOC1* expression level significantly increased. Similarly, the JA biosynthesis gene *LOX2* is directly activated by transcription factors. In vitro experiments have determined that defects in JA or JA signaling lead to delayed aging in plants, again proving that JA can induce leaf senescence [41,42].

The preservation quality of vegetable products after harvest is important. Studies have shown that MeJA is widely used in postharvest horticultural products. The application of exogenous MeJA promotes endogenous JA biosynthesis in plants, accelerates the degradation of chlorophyll, and leads to the yellowing of broccoli [43]. In the relevant literature, it has been stated that the pepper (*Capsicum annuum* L.) fruit is very sensitive to temperatures below 10 °C, which easily browns the seeds. It has also been reported that JA biosynthesis and signal transduction are involved in regulating the resistance of pepper fruit to low temperatures [44]. The effects of JA seed treatment on *Brassica juncea* L. seedlings were reported, and it was found that after imidacloprid (IMI) application and JA treatment, the chlorophyll content gradually increased [45].

The above results show that, in vegetable production, we can obtain the required products by controlling the JA concentration and application amount so as to obtain vegetable products that better meet the market demand.

### 4.2. The Influence of External Factors

JA not only participates in plant growth and development but also activates the plant defense response to adverse external environments [46]. By changing the JA content levels, some external factors can regulate the physiological responses of plants, such as their responses to mechanical damage, ozone and ultraviolet radiation, and arid environments.

These regulatory reactions are all involved in the JA signal transduction pathways. During plant growth and development, when plants are subjected to mechanical injury, their JA contents increase in response to wound or water stress, and gene expressions related to wound or water stress induction are activated [47]. The endogenous JA content in uninjured tuber tissues is generally low; however, after injury, the JA content in wound tissues reaches more than five times the original and then gradually decreases to the basic level [48]. In addition, the expressions of JA biosynthesis genes (*StAOS2*, *StAOC*, and *StOPR3*) are upregulated 2–4 h after injury.

Studies have shown that mitogen-activated protein kinase (MAPK/MPK) is involved in wound and JA signaling pathways. WIPK (wound-induced protein kinase), a direct homolog of AtMPK3, is involved in ozone resistance in plants [49]. In addition, AtMPK4 is also necessary for JA-response gene expression [50]. These results indicate that JA signal transduction is also activated in a series of ozone-induced reactions to some extent. Ozone is a strong oxidizing gas that can harm plants by entering their leaves through stomata [51]. In previous studies, MeJA treatment enabled tobacco to achieve ozone tolerance, and JA can inhibit ethylene-dependent lesions caused by ozone and induce the closure of plant stomata to resist the harm of ozone to plants [52].

Ultraviolet light is composed of UV-C (below 280 nm), UV-B (280–320 nm), and UV-A (320–390 nm). Exposure to low UV-B levels initiates signaling through UVR8 and induces secondary metabolite genes involved in UV protection. Some studies have reported that the genes involved in the MAPK cascade contribute to the resistance of plants to ultraviolet radiation [53]. UV-B is a source of stress, but it also plays a certain regulatory role in plants. The UV-B stress response is mainly due to its high dose state in non-adaptive plants, while it can induce photomorphogenic effects at low doses [54]. UV-B can damage plant DNA, thereby affecting plant growth and development, and, in response, plants have evolved various defense mechanisms. Recently, the *UV RESISTANCE LOCUS 8* (*UVR8*) gene was identified as a specific UV-B receptor in plants, suggesting that plants actively sense UV-B and regulate the downstream signaling pathways of related hormones, such as the JA signaling pathway [55]. Studies have shown that the JA and JA-Ile contents in plants increase under high-intensity UV-B conditions [56].

In summary, JA has a certain impact on how plants cope with the harm of the external environment and can induce the expressions of a series of related genes to protect plants from the infestation of external adverse environmental factors.

### 4.3. Molecular Regulation

#### 4.3.1. Synthase Regulation in Signal Transduction

The main synthetases involved in the JA biosynthesis pathway are lipoxygenase (LOX), allene oxide synthase (AOS), and allene oxide cyclase (AOC). LOX plays an important role in seed germination [57]. Moreover, when plants are subjected to severe environmental hazards (such as ultraviolet light, extreme temperatures, and insect and pathogen attacks), *LOX* gene expression is induced, resulting in an increase in the JA content [58]. The mechanical injury causes the activation of key enzymes in the synthesis of JA and MeJA, causing JA and MeJA to accumulate in the wound. The aggregated JA further expresses protective genes, including protease inhibitor genes [59].

A large number of studies have shown that JA plays an important role in regulating sesquiterpenoid metabolism, and can induce the expressions of terpenoid synthase genes and compound synthesis and release. The terpene synthase family is a medium-sized gene family [60]. In previous studies, TPS produced terpenoids by converting acyclic isoprene diphosphate into hydrocarbons. In addition, exogenous MeJA induced the release of volatile terpene compounds, suggesting that JA can improve plant resistance to fungi via the synthesis of terpene compounds [61,62]. In terms of cell components, MeJA treatment results in the up- or downregulation of the expressions of some proteins. In terms of molecular function, MeJA treatment affects the expression levels of several enzymes, including olkeone reductase, cytochrome P450 monooxygenase, and 3β-hydroxysterol dehydrogenase [63].

#### 4.3.2. Transcription Factors Regulation

The effect of the regulation of plant hormones on biological and abiotic stress responses is inseparable from that of transcription factors, which play an important role in JA signal transduction. The most active compound of JAs is (+) -7-iso-JA-Ile, a JA conjugate to isoleucine. In a study on JA-Ile, Daoxin Xie’s lab and Chuanyou Li’s lab found COI1 (JAI1) and SCF, activators (MYCs and MYBs) and repressors (JAV1 and BHlH IIId’) of JA gene expression regulation [21]. It has been reported that exogenous JA can inhibit flowering in Arabidopsis by regulating the MYC2, MYC3, and MYC4 functions [64].

The JAZ repressor binds to MYC2 to inhibit its transcriptional activation of downstream genes. Under stress conditions, endogenous levels of JA-ILE are activated to a large extent, which is sensed by the JA receptor COI1. Then, SKP1/CULLIN/F-box (SCF) COI1 binds to JAZ through the 26S proteasome pathway for ubiquitination and degradation, leading to the release of downstream transcription factors (TFs) such as MYC and JA response activation [65]. MYC2 acts downstream of the SCF^COI1^ co-receptor complex, acting as a regulatory hub in plant hormone signaling by integrating various endogenous and exogenous plant growth and development signals [66]. It has been found that JA signal transduction inhibits root growth. *JAZ4*, *JAZ8*, *JAZ9*, and *JAZ13* overexpression results in JA insensitivity, which reduces the inhibition of JA on plant root growth [67]. At present, resistance induced by MeJA or JA mainly focuses on the downstream signaling factors and reactions. Among them, MYC2 is the main transcription factor of the JA signaling pathway. Shu and his colleagues compared *SlMYC2* mutants with wild-type plants and found that the *SlMYC2* knockouts had significantly reduced disease defense enzyme and antioxidant enzyme activities. Meanwhile, the expressions of key genes related to the JA biosynthesis and signal transduction pathway were decreased [68]. LeMYC2 positively regulates root growth in tomato plants [69]. In addition, MYB is the most sensitive gene to JA signals, followed by *AP2-EREBP*, *C3H*, and *WRKY* [70].

## 5. The Role of JA in Vegetable Crop Resistance

### 5.1. Disease and Pest Resistance

Crop pathogens and pests seriously affect the quality and yields of agricultural products [71]. Worldwide, about 40 percent of crop yields are affected by pests and diseases [72]. At present, resistance induced via MeJA or JA mainly focuses on the downstream signaling factor and reactions [68]. According to reports, the WRKY TF has proven to be important in responding to pathogen attacks, and *PpWRKY45* and *PpWRKY70* are related to the resistance reaction toward stolon induced by MeJA through JA biosynthesis activation [73,74]. By comparing the normal plants and potato plants with an *St13-AOS* gene knockout, it was found that the JA contents in the plants with the *St13-AOS* gene knockout were significantly lower than those in the normal plants, the activation degree of the wound reactivity gene was decreased, and the potato soft-rot symptom severity was increased [75]. Moreover, *IbBBX24* overexpression was found to significantly increase the blight occurrence in sweet potato, and IbBBX24 regulates the JA pathway by antagonizing the JA signal inhibitor IbJAZ10 while reducing the inhibitory effect of IbJAZ10 on the JA signal activator IbMYC2 [76]. In conclusion, JA signaling plays an important role in improving vegetable disease resistance. These results indicate that the JA signal transduction pathway has a certain resistance to plant pathogens. In addition, the primary JA function is the promotion of the production of secondary metabolites. In tomato, systemin overexpression leads to JA accumulation, which improves the insect resistance of the plant [77]. After an insect injury, exogenous MeJA induces endogenous signals at the wound site. It has been reported that spraying MeJA on tomatoes and cabbage significantly reduces the ability of *Spodoptera litura* larvae to grow [78]. After the MeJA pretreatment of the seeds of *Lupinus mutabilis* Sweet, only the seeds treated with MeJA showed the ability to damage insects. It was also revealed that the use of MeJA upregulated the activities of the JA biosynthesis genes *LmLOX2* and *LmAOS*, as well as those of jasmonic acid-related defense genes (*LmTPS1*, *LmTPS4*, *LmPI2*, *LmMBL*, *LmL/ODC*, *LmCSD1*, and *LmPOD*) [79]. These results suggest that MeJA, as an inducer, may induce the defense mechanism of plants against pathogens and insects by regulating the biosynthesis of terpenoids, protease inhibitors, and antioxidant enzyme activities. They also prove the importance of JA to the growth and quality of vegetables.

### 5.2. Abiotic Stress

At present, the main abiotic stress reactions encountered in vegetable production are salt stress, drought stress, high- and low-temperature stress, and heavy metal stress. These stresses can have negative impacts on the quality of vegetables and their yields. Studies have shown that MeJA has a certain easing effect on plant stress damage. At the same time, as an endogenous signaling molecule, MeJA is involved in plant stress resistance under salt stress, drought stress, high- and low-temperature stress, and heavy metal stress. In addition, MeJA can reduce the adverse effects of transport stress on broccoli by increasing its vitamin C content, inhibiting reactive oxygen species accumulation, and inducing antioxidant gene expression and enzyme activities [80].

#### 5.2.1. Salt Stress

Salt stress mainly refers to osmotic stress and the ion toxicity of plant root cells in high-salt/-alkaline environments. It is one of the important abiotic stresses affecting vegetable production, as most vegetables are sensitive to soil salt. Relevant research shows that the malondialdehyde (MDA) level increases and the chlorophyll content decreases in pepper under salt stress, and an important transcription factor, CaMADS, which is induced by MeJA, is involved in pepper’s response to salt stress [81]. It was reported that endogenous JA enhanced the tomato salt tolerance mainly by maintaining the balance of reactive oxygen species in vivo. Exogenous JA treatment can improve the photosynthetic rate, proline content, ABA level, and antioxidant enzyme activity [82].

The report found that MeJA could significantly improve the saline–alkali stress tolerance of tomatoes. It was found that the transcription factor *SlWRKY80* could actively regulate the resistance of tomato to saline–alkali stress. The application of exogenous MeJA sprayed on *SlWRKY80* knockout plants decreased their sensitivity to saline–alkali stress [83]. Among them, *SlWRKY80* mainly binds to the related promotor and positively regulates its transcription, promoting the synthesis of spermidine and Na^+^/K^+^ homeostasis and achieving the active regulation of saline–alkali stress. In addition, when JA was applied to potatoes under normal and high-salt conditions, the results showed that it had a significant protective effect on the potato plants under salt stress [84]. JA partially abolished the negative salt effect on the main photosynthetic pigments and maintained the cell’s osmotic status during salinization. In addition, JA significantly improved the salt tolerance of cucumber by increasing the superoxide dismutase, catalase, and ascorbate peroxidase activities [85]. Moreover, under salt stress, exogenous methyl jasmonate promoted proline accumulation mainly by activating the ornithine pathway of proline biosynthesis and inhibiting the proline degradation pathway. Spraying MeJA on the surface of sweet potato leaf showed that MeJA treatment enhanced salinity stress tolerance in plants, possibly through the regulation of stomatal closure [86]. Studies have shown that methyl jasmonate reduces ion leakage and the production of malondialdehyde and hydrogen peroxide but increases the contents of photosynthetic pigments, soluble carbohydrates, proline, total phenols, and flavonoids [87].

#### 5.2.2. Drought Stress

Under drought conditions, plants cannot carry out normal physiological activities, water consumption is much higher than water absorption due to transpiration, the turgor pressure of plant cells is reduced, and physiological activities are inhibited [88,89]. In the absence of human interference, plants will tolerate drought by significantly reducing their leaf areas, root elongation, and branching angles. JA has been shown to improve plant performance and regulate the stomatal status in plants under drought conditions. In the absence of water, the JAZ protein degrades and activates related transcription factors (such as MYC2), which then stimulate the upregulation of stress tolerance genes [90]. Research shows that the cassava SPL9 negatively regulates crop drought resistance, and rMeSPL9-SRDX overexpression lines showed increased proline and anthocyanin contents as well as endogenous JA and soluble sugar accumulations [91]. The results indicate that drought conditions led to JA biosynthesis. The application of exogenous JA further proved that JA had better drought resistance and promoted the stomatal closure of cassava leaves. Therefore, exogenous JA application can significantly alleviate the harm caused by drought by improving the photosynthetic rate, stomatal conductance, the transpiration rate, and antioxidant defense energy.

#### 5.2.3. High- and Low-Temperature Stress

Among the abiotic stresses, temperature stress is an unpredictable problem leading to crop production issues, and frost and high summer temperatures are possible causes of yield reductions. In order to resist environmental temperature changes, plants have developed adaptation mechanisms, and plant hormones also play an important role [92,93]. Under high-temperature conditions, the accumulation of JA and its active substance JA-Ile increases significantly, indicating that JA plays a role in the reaction [86]. Recently, studies have shown that topical MeJA can drive miRNAs that target heat-resistant genes in plants, thereby achieving plant resistance to high temperatures [94,95]. Moreover, some scholars have observed that the H_2_O_2_ content in tomatoes changes with the low-temperature stress response and MeJA supplementation, indicating that MeJA protects plants by controlling the H_2_O_2_ and TBARS levels under low-temperature conditions [96]. JA can also resist or alleviate chilling injury by increasing the soluble sugar content and regulating reactive oxygen species (ROS) [97,98]. In the fruits of *Capsicum annuum*, the expression levels of *CaERF1*, *CaERF3-1*, *CaERF5*, and *CaERF10* in ERF family genes regulated by JA were significantly increased under cold storage [44].

#### 5.2.4. Heavy Metal Stress

Heavy metals (HMs) not only harm the health of plants but also endanger the safety of human beings [99]. HMs may damage the nucleic acid and enzyme structures in plants, preventing the absorption of key metals and leading to nutrient deficiencies and imbalances in the plant [100]. Among them, lead (Pb), cadmium (Cd), nickel (Ni), manganese (Mn), and other heavy metals have strong plant toxicities. The fresh weights and photosynthetic pigment concentrations of plants exposed to the abovementioned heavy metals are reduced [101]. Yan and his colleagues found that MeJA could significantly reduce the damage of Cd to pepper seedlings [102]. MeJA controls the heavy metal Cd damage to plants by reducing malondialdehyde (MDA) and H_2_O_2_ and enhances the antioxidant enzyme activity and phenolic content [103]. Exogenous JA alleviates plant toxicity caused by chromium (Cr) by affecting photosynthetic pigments and the gas-exchange parameters and enhances the antioxidant enzyme, ascorbic acid, and glyoxalase activities to induce plant resistance systems [104]. The genes of *LOX1* and *OPR1* were upregulated and the *JAR1* level was increased in the roots of pea seedlings grown under a sublethal dose of lead, which was the result of the JA signal transduction from the leaves to the roots in response to the stress response [105].

## 6. Quality

Methyl jasmonate can regulate the production of bioactive substances in plants and cause the accumulation of plant aromatic substances. At the same time, it can regulate the postharvest fruit quality and stress response by regulating plant antioxidant systems, such as phenols and antioxidant-related enzymes. In addition, the browning reaction that often occurs during vegetable processing can lead to declines in the appearance, flavor, and nutritional quality of vegetable products. The main cause of browning is the formation of quinones by substrates such as phenols, anthocyanins, and flavonoids. A study by Wang and his colleagues showed that MeJA significantly increased the soluble sugar, protein, enzymatic pyruvate, sulfide, and aromatic volatile compound contents of Chinese leek and improved its characteristic flavor [106].

### 6.1. Vitamin C

Vitamin C, also known as L-ascorbic acid, is a water-soluble vitamin with free radical scavenging, antioxidant, and other effects. Ascorbic acid is the first line of defense against damaging reactive oxygen species (ROS) and helps to protect plant cells from many factors that induce oxidative stress, including wounds, ozone, high salinity, and pathogen attacks. However, the defense of these stress responses also depends on JA signal transduction [107]. The hormonal regulation of vitamin C biosynthesis in plants revealed by B. A. Wolucka suggests that methyl jasmonate treatment enhances vitamin C biosynthesis [108]. JA activates oxidative stress defense responses, such as the ascorbic acid and glutathione metabolic pathways, induces the accumulation of ascorbate, and increases dehydroascorbate reductase activity [109]. MeJA treatment can significantly increase the antioxidant activity and vitamin C contents of leek leaves, and methyl jasmonate treatment can inhibit the decrease in vitamin C in blueberry fruits [106,110]. Studies have shown that methyl jasmonate can reduce the adverse effects of transport stress on broccoli by increasing its vitamin C content, inhibiting reactive oxygen species accumulation, and inducing antioxidant gene expression and enzyme activity. According to reports, scholars studied two Arabidax knockout mutants (AO and vtc1) and found that JA, as a secondary signal in response to viral infection, may play an important role in the accumulation of endogenous ascorbic acid (AS) [111]. MeJA can effectively increase the vitamin C contents in vegetables and inhibit decline.

### 6.2. Flavonoid Substances

Flavonoid compounds affect the quality, economic value, color, and aroma of fruits and vegetables. In addition, flavonoids are natural polyphenols with antioxidant, antibacterial, and anti-inflammatory properties [112]. One study indicated that MeJA may upregulate the expressions of upstream genes and downregulate the expressions of downstream genes in the flavonoid biosynthesis pathway, thereby promoting quinone chalcone biosynthesis [113]. Among the flavonoid substances, anthocyanins are important antioxidants for health promotion. The combined treatment of methyl jasmonate/sucrose had a certain effect on anthocyanin production in grapes, which effectively stimulated the expressions of stilbene synthase, flavono-o-glucosyltransferase, and protease inhibitor genes and triggered anthocyanin accumulation in the cells [110]. It was reported that the application of exogenous MeJA enhanced the anthocyanin accumulation in red meat apple callus, as the related *MdCHS*, *MdF3H*, and *MdUFGT* expressions were upregulated [114]. By applying MeJA to eggplant, the JA reaction factor SmMYB5 was a positive regulator of anthocyanins. Low light conditions affect anthocyanin synthesis in eggplant skins. After MeJA application, SmMYB5 in plant cells interacts with the anthocyanin-promoted bHLH transcription factor SmTT8 to form a complex, which directly activates the anthocyanin synthase promoter [115]. In addition to genes related to anthocyanin biosynthesis, the expressions of key genes involved in procyanidin biosynthesis (PcANR2a and PcLAR1) are also upregulated by MeJA.

### 6.3. Phenolic Compounds

Phenolic compounds widely exist in nature, and most of them have aroma and antioxidant activities, which are mainly found in the fruits, skins, roots, leaves, and other tissues and organs of plants. Different plants contain different phenolic compounds, which are mainly divided into flavonoids and non-flavonoids. Phenolic compounds are effective non-enzymatic protectors against oxidative stress, and they can act as antioxidant enzymes in a variety of ways [116]. It has been reported that phenol biosynthesis in carrots increases when they are stressed by wounds or ultraviolet light. The signaling molecules that trigger this reaction are ET and LOX, among which LOX is a key enzyme in JA biosynthesis [117]. Therefore, we can assume that the JA signal transduction pathway is involved in phenol activation and accumulation. Under the stress response, the level of total phenolic compounds in tomato plants increased after JA treatment [118]. The results indicated that JA enhanced tomato tolerance mainly by maintaining reactive oxygen species (ROS) homeostasis. Studies have shown that the phenol level of kale treated with MeJA was higher compared with the control group, which indicates that the kale treated with MeJA can be used as a functional food for fresh consumption [119]. Studies show that an appropriate MeJA treatment concentration can increase the phenolic compound content in leek leaves. The romaine lettuce (*Lactuca sativa* L.) treated with exogenous MeJA showed significantly increased total phenolic compound contents and antioxidant capacity [120]. The results showed that the use of JA is helpful to improve the nutritional value of vegetable crops.

### 6.4. Terpenoids

Terpenoids are a kind of isoprene compound and the main component of plant flavor, resin, and pigment. MeJA treatment induces an important intermediate of the MEP pathway, 2-C-methyl-D-erythritol 2, 4-cyclodiphosphate, and most enzymes related to the biosynthesis of terpenoid skeletons are significantly upregulated under the MeJA treatment [63]. The accumulation of plant terpenoids under cold stress is mainly via the expression levels and activities of endogenous plant hormones (such as JA), important transcription factors (such as bHLH and those of the MYB family), and the expressions and activities of the key enzyme genes in biosynthetic pathways [121].

The *AarbHLH* gene expression profile in Artemisinin treated with MeJA was analyzed. It was found that *AarbHLH* was mainly expressed in the leaves and stems and was less expressed in the roots. In addition, under the MeJA treatment, *AarbHLH* genes belonging to the MYC class were upregulated, which was consistent with the terpenoids change trend [122]. Studies have shown that the use of MeJA can promote terpenoid synthesis and accumulation in most plants. MYC2 and MYC4 are important regulators of the JA signaling and terpenoid biosynthesis pathways. In *Curcuma wenyujin*, MeJA induced JA synthesis and signal transduction genes to activate the JA signaling pathway, while downstream JA response genes upregulated the expression levels of iron terpene synthesis genes, thereby promoting terpene biosynthesis [12]. The above studies have proven that the JA signaling pathway has a certain regulatory relationship with terpene compound biosynthesis, and the effect of the MeJA treatment on the content of terpene compounds is mainly from the regulation of their biosynthesis-related genes.

### 6.5. Lignin

Lignin, one of the main components of plant cell walls, is a natural phenolic polymer. The presence of lignin is beneficial to plant growth, tissue, and organ development and has an impact on plant lodging resistance and some biological and abiotic stress responses [123]. JA is involved in lignin biosynthesis and regulates the lignin level to a certain extent [124]. The MeJA treatment can improve the hardness and anthocyanins of fruits, and the lignin content is higher than that of untreated fruits and the rot rate is lower. The fruit shows better antioxidant properties and has a longer shelf life [125]. It has been previously reported that JA can induce CAD gene expression and co-promote lignin deposition with reactive oxygen species [126]. In addition, the changing trend of the JA contents in mature and senescent tissues was positively correlated with the lignification of plants [127]. By observing *RESPIRATORY BURST OXIDASE HOMOLOG D (RBOHD)* induced by JAR1 mutant seedlings, it was found that reactive oxygen species (ROS) and the JA-based signaling process played roles in the regulation of the CWD-induced lignin deposition [124]. It has been reported that when JA was sprayed on the hypocotyl of young cannabis plants, changes were found in the synthesis of the lignin monomers in the hypocotyls of the cannabis plants, and the treated hypocotyls had additional secondary phloem fibers, indicating that JA promoted the synthesis of lignin monomers and increased lignin deposition [128].

## 7. Future Perspectives

The plant hormone JA is an important signaling molecule that regulates plant growth and development and the ability of plants to cope with adversity. However, in many vegetable crops, JA accumulates in relatively small amounts, which may affect plant resistance and quality. Therefore, we need to explore JA synthesis and extraction in plants to enhance the resistance and quality of vegetable crops.

In order to achieve the synthesis and extraction of JA in vivo and in vitro in plants, we can use biotechnological means. One method is to introduce the genes related to the JA synthetic pathway into vegetable crops through transgenic technology to increase the JA accumulation in plants. The other method is to use gene-editing technology to accurately improve the genes related to the JA synthesis pathway in plants to improve their ability to synthesize JA. These methods can help us achieve increased JA accumulation in vegetable crops, thereby improving plant resistance and quality.

The JA accumulation in different vegetable crops may be different, which may affect their development regulation and stress resistance. Therefore, we need to further understand the JA signaling pathway in different vegetable crops and understand its role in plant development regulation and signaling. By studying the JA signaling pathways in different vegetable crops, we can better understand their growth, development, and resistance mechanisms, and can provide a basis for improving vegetable crops according to these mechanisms in the future.

In the future, we can use new technologies, such as transgenic and gene editing, to synthesize JA and apply it to the resistance and quality improvement of vegetable crops. Through these new techniques, we can precisely regulate the JA synthesis in plants to improve their resistance abilities so that they can better adapt to various adverse environments. In addition, the use of new technology for JA synthesis can also improve the quality of vegetable crops, making them more high-quality and nutrient-rich to meet the growing needs of people. Therefore, JA synthesis using new technology has important significance and potential application prospects for the resistance of vegetable crops and improvements in their quality.

## Data Availability

Data are contained within the article.
